# A review on laboratory liver function tests

**Published:** 2009-11-22

**Authors:** Shivaraj Gowda, Prakash B. Desai, Vinayak V. Hull, Avinash A K. Math, Sonal N. Vernekar, Shruthi S. Kulkarni

**Affiliations:** 1 Dept of Biochemistry, J. N. Medical College, Belgaum 590010. Karnataka. India

**Keywords:** Laboratory liver test, bilirubin, alanine amino transferase, aspartate amino transferase, ratio of aminotransferases, alkaline phosphatase, gamma glutamyl transferase, 5′ nucleotidase, ceruloplasmin, α-fetoprotein

## Abstract

Laboratory liver tests are broadly defined as tests useful in the evaluation and treatment of patients with hepatic dysfunction. The liver carries out metabolism of carbohydrate, protein and fats. Some of the enzymes and the end products of the metabolic pathway which are very sensitive for the abnormality occurred may be considered as biochemical marker of liver dysfunction. Some of the biochemical markers such as serum bilirubin, alanine amino transferase, aspartate amino transferase, ratio of aminotransferases, alkaline phosphatase, gamma glutamyl transferase, 5′ nucleotidase, ceruloplasmin, α-fetoprotein are considered in this article. An isolated or conjugated alteration of biochemical markers of liver damage in patients can challenge the clinicians during the diagnosis of disease related to liver directly or with some other organs. The term “liver chemistry tests” is a frequently used but poorly defined phrase that encompasses the numerous serum chemistries that can be assayed to assess hepatic function and/or injury.

## Laboratory Liver Tests

### Serum Bilirubin

Bilirubin is the catabolic product of haemoglobin produced within the reticuloendothelial system, released in unconjugated form which enters into the liver, converted to conjugated forms bilirubin mono and diglucuronides by the enzyme UDP-glucuronyltransferase [1]. Normal serum total bilirubin varies from 2 to 21μmol/L. The indirect (unconjugated) bilirubin level is less than 12μmol/L and direct (conjugated) bilirubin less than 8μmol/L [2]. The serum bilirubin levels more than 17μmol/L suggest liver diseases and levels above 24μmol/L indicate abnormal laboratory liver tests [3, 4]. Jaundice occurs when bilirubin becomes visible within the sclera, skin, and mucous membranes at a blood concentration of around 40 μmol/L [5]. The occurrence of unconjugated hyperbilirubinemia due to over production of bilirubin, decreased hepatic uptake or conjugation or both. It is observed in genetic defect of UDP-glucuronyltransferase causing Gilbert\'s syndrome, Crigler-Najjar syndrome and reabsorption of large hematomas and ineffective erythropoiesis [6, 7]. In viral hepatitis, hepatocellular damage, toxic or ischemic liver injury higher levels of serum conjugated bilirubin is seen. Hyperbilirubinemia in acute viral hepatitis is directly proportional to the degree of histological injury of hepatocytes and the longer course of the disease [3]. It has been observed that the decrease of conjugated serum bilirubin is a bimodal fashion when the biliary obstruction is resolved [8]. Parenchymal liver diseases or incomplete extrahepatic obstruction due to biliary canaliculi give lower serum bilirubin value than those occur with malignant obstruction of common bile duct but the level remains normal in infiltrative diseases like tumours and granuloma [9]. Raised Serum bilirubin from 20.52 μmol/L to 143.64μmol/L in acute inflammation of appendix has been observed [10]. In normal asymptomatic pregnant women total and free bilirubin concentrations were significantly lower during all three trimesters and a decreased conjugated bilirubin was observed in the second and third trimesters [11]. The recent study has shown that a high serum total bilirubin level may protect neurologic damage due to stroke [12].

### Alanine amino transferase (ALT)

ALT is found in kidney, heart, muscle and greater concentration in liver compared with other tissues of the body. ALT is purely cytoplasmic catalysing the transamination reaction [1]. Normal serum ALT is 7–56 U/ L [2]. Any type of liver cell injury can reasonably increases ALT levels. Elevated values up to 300 U/L are considered nonspecific. Marked elevations of ALT levels greater than 500 U/L observed most often in persons with diseases that affect primarily hepatocytes such as viral hepatitis, ischemic liver injury (shock liver) and toxin-induced liver damage. Despite the association between greatly elevated ALT levels and its specificity to hepatocellular diseases, the absolute peak of the ALT elevation does not correlate with the extent of liver cell damage [13]. Viral hepatitis like A, B, C, D and E may be responsible for a marked increase in aminotransferase levels. The increase in ALT associated with hepatitis C infection tends to be more than that associated with hepatitis A or B [14]. Moreover in patients with acute hepatitis C serum ALT is measured periodically for about 1 to 2 years [1]. Persistence of elevated ALT for more than six months after an occurrence of acute hepatitis is used in the diagnosis of chronic hepatitis. Elevation in ALT levels are greater in persons with nonalcoholic steatohepatitis than in those with uncomplicated hepatic steatosis [15]. In a recent study the hepatic fat accumulation in childhood obesity and nonalcoholic fatty liver disease causes serum ALT elevation. Moreover increased ALT level was associated with reduced insulin sensitivity, adiponectin and glucose tolerance as well as increased free fatty acids and triglycerides [16]. Presence of Bright liver and elevated plasma ALT level was independently associated with increased risk of the metabolic syndrome in adults [17]. ALT level is normally elevated during 2nd trimester in asymptomatic normal pregnancy [11]. In one of the study, serum ALT levels in symptomatic pregnant patients such as in hyperemesis gravidarum was 103.5U/L, in pre-eclampsia patients was 115U/L and in haemolysis with low platelet count patients showed 149U/L. However in the same study ALT rapidly drops more than 50% of the elevated values within 3 days indicating the improvement during postpartum [4]. One of the recent study has shown that coffee and caffeine consumption reduces the risk of elevated serum ALT activity in excessive alcohol consumption, viral hepatitis, iron overload, overweight, and impaired glucose metabolism [18].

### Aspartate amino transferase (AST)

AST catalyse transamination reaction. AST exist two different isoenzyme forms which are genetically distinct, the mitochondrial and cytoplasmic form. AST is found in highest concentration in heart compared with other tissues of the body such as liver, skeletal muscle and kidney [1]. Normal serum AST is 0 to 35U/L [2]. Elevated mitochondrial AST seen in extensive tissue necrosis during myocardial infarction and also in chronic liver diseases like liver tissue degeneration and necrosis [3]. About 80% of AST activity of the liver is contributed by the mitochondrial isoenzyme, whereas most of the circulating AST activity in normal people is derived from the cytosolic isoenzyme [3]. However the ratio of mitochondrial AST to total AST activity has diagnostic importance in identifying the liver cell necrotic type condition and alcoholic hepatitis [19]. AST elevations often predominate in patients with cirrhosis and even in liver diseases that typically have an increased ALT [20]. AST levels in symptomatic pregnant patient in hyperemesis gravidarum were 73U/L, in pre-eclampsia 66U/L, and 81U/L was observed in hemolysis with low platelet count and elevated liver enzymes [4].

### AST/ALT ratio

The ratio of AST to ALT has more clinical utility than assessing individual elevated levels. A coenzyme pyridoxal-5\'-phosphate deficiency may depress serum ALT activity and consequently increases the AST/ALT ratio [21, 22]. The ratio increases in progressive liver functional impairment and found 81.3% sensitivity and 55.3% specificity in identifying cirrhotic patients [23]. Whereas mean ratio of 1.45 and 1.3 was found in alcoholic liver disease and post necrotic cirrhosis respectively [24]. The ratio greater than 1.17 was found in one year survival among patients with cirrhosis of viral cause with 87% sensitivity and 52% specificity [25]. An elevated ratio greater than 1 shows advanced liver fibrosis and chronic hepatitis C infection [26]. However, an AST/ALT ratio greater than 2 characteristically is present in alcoholic hepatitis. A recent study differentiated nonalcoholic steatohepatitis (NASH) from alcoholic liver disease showing AST/ALT ratio of 0.9 in NASH and 2.6 in patients with alcoholic liver disease. A mean ratio of 1.4 was found in patients with cirrhosis related to NASH [27]. Wilson\'s disease can cause the ratio to exceed 4.5 and similar such altered ratio is found even in Hyperthyroidism [28, 29].

### Alkaline phosphatase (ALP)

ALP is present in mucosal epithelia of small intestine, proximal convoluted tubule of kidney, bone, liver and placenta. It performs lipid transportation in the intestine and calcification in bone. The serum ALP activity is mainly from the liver with 50% contributed by bone [1]. Normal serum ALP is 41 to 133U/L [2]. In acute viral hepatitis, ALP usually remains normal or moderately increased. Elevation of ALP with prolonged itching is related with Hepatitis A presenting cholestasis. Tumours secrete ALP into plasma and there are tumour specific isoenzymes such as Regan, Nagao and Kasahara [30]. Hepatic and bony metastasis can also cause elevated levels of ALP. Other diseases like infiltrative liver diseases, abscesses, granulomatous liver disease and amyloidosis may cause a rise in ALP. Mildly elevated levels of ALP may be seen in cirrhosis, hepatitis and congestive cardiac failure [30]. Low levels of ALP occur in hypothyroidism, pernicious anaemia, zinc deficiency and congenital hypophosphatasia [31]. ALP activity was significantly higher in the third trimester of asymptomatic normal pregnancy showing extra production from placental tissue [11]. ALP levels in hyperemesis gravidarum were 21.5U/L, in pre-eclampsia 14U/L, and 15U/L in haemolysis with low platelet count was seen during symptomatic pregnancy [4]. Transient hyperphosphataemia in infancy is a benign condition characterized by elevated ALP levels of several folds without evidence of liver or bone disease and it returns to normal level by 4 months [32]. ALP has been found elevated in peripheral arterial disease, independent of other traditional cardiovascular risk factors [33]. Often clinicians are more confused in differentiating liver diseases and bony disorders when they see elevated ALP levels and in such situations measurement of gamma glutamyl transferase assists as it is raised only in cholestatic disorders and not in bone diseases [30].

### Gamma Glutamyl Transferase (GGT)

GGT is a microsomal enzyme present in hepatocytes and biliary epithelial cells, renal tubules, pancreas and intestine. It is also present in cell membrane performing transport of peptides into the cell across the cell membrane and involved in glutathione metabolism. Serum GGT activity mainly attributed to hepatobiliary system even though it is found in more concentration in renal tissue [1]. The normal level of GGT is 9 to 85 U/L [2]. In acute viral hepatitis the levels of GGT will reach the peak in the second or third week of illness and in some patients remain elevated for 6 weeks [30]. Increased level is seen in about 30% of patients with chronic hepatitis C infection [34]. Other conditions like uncomplicated diabetes mellitus, acute pancreatitis, myocardial infarction, anorexia nervosa, Gullian barre syndrome, hyperthyroidism, obesity and dystrophica myotonica caused elevated levels of GGT [30]. Elevated serum GGT levels of more than 10 times is observed in alcoholism. It is partly related to structural liver damage, hepatic microsomal enzyme induction or alcoholic pancreatic damage [35]. GGT can also be an early marker of oxidative stress since serum antioxidant carotenoids namely lycopene, α-carotene, β-carotene, and β-cryptoxanthin are inversely associated with alcohol-induced increase of serum GGT found in moderate and heavy drinkers [36]. GGT levels may be 2–3 times greater than the upper reference value in more than 50% of the patients with nonalcoholic fatty liver disease [37]. There is a significant positive correlation between serum GGT and triglyceride levels in diabetes and the level decreases with treatment especially when treated with insulin. Whereas serum GGT does not correlate with hepatomegaly in diabetes mellitus [38]. Serum GGT activity was significantly lower in the second and third trimesters of normal asymptomatic pregnancy [11]. The levels of GGT in hyperemesis gravidarum was 45U/L, in pre-eclampsia 17U/L, and 35U/L in hemolysis with low platelet count and elevated liver enzymes was found during symptomatic pregnancy [4]. The primary usefulness of GGT is limited in ruling out bone disease as GGT is not found in bone [30].

### 5′ Nucleotidase (NTP)

NTP is a glycoprotein generally disseminated throughout the tissues of the body localised in cytoplasmic membrane catalyzing release of inorganic phosphate from nucleoside-5-phosphates. The normal range established is 0 to 15U/L [1]. Raised levels of NTP activity were found in patients with obstructive jaundice, parenchymal liver disease, hepatic metastases and bone disease [9]. NTP is precise marker of early hepatic primary or secondary tumours. ALP levels also increased in conjugation with NTP showing intra or extra hepatic obstruction due to malignancy [39]. Elevation of NTP is found in acute infective hepatitis and also in chronic hepatitis [40]. In acute hepatitis elevation of NTP activity is more when compared with chronic hepatitis and it is attributed to shedding of plasma membrane with ecto NTP activity due to cell damage, or leakage of bile containing high NTP activity [41]. Serum NTP activity was slightly but significantly higher in the second and third trimesters of pregnancy [11].

### Ceruloplasmin

Ceruloplasmin is synthesized in the liver and is an acute phase protein. It binds with the copper and serves as a major carrier for copper in the blood [1]. Normal plasma level of ceruloplasmin is 200 to 600mg/L [2]. The level is elevated in infections, rheumatoid arthritis, pregnancy, non Wilson liver disease and obstructive jaundice. Low levels may also be seen in neonates, menke’s disease, kwashiorkor, marasmus, protein losing enteropathy, copper deficiency and aceruloplasminemia [3]. In Wilson\'s disease ceruloplasmin level is depressed. Decreased rate of synthesis of the ceruloplasmin is responsible for copper accumulation in liver because of copper transport defect in golgi apparatus, since ATP7B is affected [30]. Serum ceruloplasmin levels were elevated in the chronic active liver disease (CALD) but lowered in the Wilson’s disease (WD). Hence it is the most reliable routine chemical screening test to differentiate between CALD and WD [42].

### α-fetoprotein (AFP)

The AFP gene is highly activated in foetal liver but is significantly repressed shortly after birth. The mechanisms that trigger AFP transcriptional repression in postpartum liver are not properly understood. AFP is the major serum protein in the developing mammalian foetus produced at high levels by the foetal liver and visceral endoderm of the yolk sac and at low levels by foetal gut and kidney. AFP is required for female fertility during embryonic development by protecting the developing female brain from prenatal exposure to estrogen [43]. In response to liver injury and during the early stages of chemical hepatocarcinogenesis led to the conclusion that maturation arrest of liver-determined tissue stem cells give rise to hepatocellular carcinomas [44]. The normal level of AFP is 0 to 15μg/L [2]. An AFP value above 400 – 500μg/L has been considered to be diagnostic for hepatocellular carcinoma (HCC) in patients with cirrhosis. A high AFP concentration ≥ 400μg/L in HCC patients is associated with greater tumour size, bilobar involvement, portal vein invasion and a lower median survival rate [45]. Higher serum AFP levels independently predict a lower sustained virological response (SVR) rate among patients with chronic hepatitis C [46]. There are three different AFP variants, differing in their sugar chains (AFP-L1, AFP-L2, AFP-L3). AFP-L1, the non- Lens culinaris agglutinin (LCA) -bound fraction, is the main glycoform of AFP in the serum of patients with non-malignant chronic liver disease. In contrast, Lens culinaris-reactive AFP, also known as AFP-L3, is the main glycoform of AFP in the serum of HCC patients and it can be detected in approximately one third of patients with small HCC (< 3 cm), when cut-off values of 10% to 15% are used [47]. AFP-L3 acts as a marker for clearance of HCC after treatment. It is reported that an AFP-L3 level of 15% or more is correlated with HCC- associated portal vein invasion [48]. Estimating the AFP-L3 / AFP ratio is helpful in diagnosis and prognosis of HCC [49]. There is a direct association between second-trimester maternal serum alpha-fetoprotein levels and the risk of sudden infant death syndrome (SIDS), which may be mediated in part through impaired foetal growth and preterm birth [50].

## Conclusion

Laboratory liver tests help to elucidate the alteration of markers which reflect the liver disease. The assessment of enzyme abnormalities like, the predominant pattern of enzyme alteration, the magnitude of enzyme alteration in the case of aminotransferases, isolated elevation or in conjugation with some other parameter, the rate of change and the nature of the course of alteration or follow up of 6 months to 1–2 years helps in the diagnosis of the disease. But a single laboratory liver test is of little value in screening for liver disease as many serious liver diseases may be associated with normal levels and abnormal levels might be found in asymptomatic healthy individuals. The pattern of enzyme abnormality, interpreted in the context of the patient’s symptoms can aid in directing the subsequent diagnosis.

## List of abbreviations

AFP: α-fetoprotein
ALP: Alkaline phosphatase
ALT: Alanine amino transferase
AST: Aspartate amino transferase
CALD: Chronic active liver disease
GGT: Gamma Glutamyl Transferase
HCC: Hepatocellular carcinoma
LCA: Lens culinaris agglutinin
NAHS: Nonalcoholic steatohepatitis
NTP: 5′ Nucleotidase
SIDS: Sudden infant death syndrome
SVR: Sustained virological response
UDP: Uridyne diphosphate
WD: Wilson’s disease
